# Subcellular Localization and Functional Characterization of GII.4 Norovirus-Encoded NTPase

**DOI:** 10.1128/JVI.01824-17

**Published:** 2018-02-12

**Authors:** Ju-Bei Yen, Ling-Huei Wei, Lee-Wen Chen, Li-Yu Chen, Chien-Hui Hung, Shie-Shan Wang, Pey-Jium Chang

**Affiliations:** a Graduate Institute of Clinical Medical Sciences, College of Medicine, Chang-Gung University, Taoyuan, Taiwan; b Department of Pediatrics, Chang-Gung Memorial Hospital, Chiayi, Taiwan; c Department of Respiratory Care, Chang-Gung University of Science and Technology, Chiayi, Taiwan; d Department of Pediatric Surgery, Chang-Gung Memorial Hospital, Chiayi, Taiwan; e School of Traditional Chinese Medicine, College of Medicine, Chang-Gung University, Taoyuan, Taiwan; f Department of Nephrology, Chang-Gung Memorial Hospital, Chiayi, Taiwan; Instituto de Biotecnologia/UNAM

**Keywords:** norovirus, NTPase, mitochondria, Nterm, P22, apoptosis

## Abstract

The genotype II.4 (GII.4) variants of human noroviruses (HuNVs) are recognized as the major agent of global gastroenteritis outbreaks. Due to the lack of an efficient cell culture system for HuNV propagation, the exact roles of HuNV-encoded nonstructural proteins (including Nterm, NTPase, P22, VPg, Pro, and RdRp) in viral replication or pathogenesis have not yet been fully understood. Here, we report the molecular characterization of the GII.4 HuNV-encoded NTPase (designated GII-NTPase). Results from our studies showed that GII-NTPase forms vesicular or nonvesicular textures in the cell cytoplasm, and the nonvesicular fraction of GII-NTPase significantly localizes to the endoplasmic reticulum (ER) or mitochondria. Deletion analysis revealed that the N-terminal 179-amino-acid (aa) region of GII-NTPase is required for vesicle formation and for ER colocalization, whereas the C-terminal region is involved in mitochondrial colocalization. In particular, two mitochondrion-targeting domains were identified in the C-terminal region of GII-NTPase which perfectly colocalized with mitochondria when the N-terminal region of GII-NTPase was deleted. However, the corresponding C-terminal portions of NTPase derived from the GI HuNV did not show mitochondrial colocalization. We also found that GII-NTPase physically interacts with itself as well as with Nterm and P22, but not VPg, Pro, and RdRp, in cells. The Nterm- and P22-interacting region was mapped to the N-terminal 179-aa region of GII-NTPase, whereas the self-assembly of GII-NTPase could be achieved via a head-to-head, tail-to-tail, or head-to-tail configuration. More importantly, we demonstrate that GII-NTPase possesses a proapoptotic activity, which can be further enhanced by coexpression with Nterm or P22.

**IMPORTANCE** Despite the importance of human norovirus GII.4 variants in global gastroenteritis outbreaks, the basic biological functions of the viral nonstructural proteins in cells remain rarely investigated. In this report, we focus our studies on characteristics of the GII.4 norovirus-encoded NTPase (GII-NTPase). We unexpectedly find that GII-NTPase can perfectly colocalize with mitochondria after its N-terminal region is deleted. However, such a phenomenon is not observed for NTPase encoded by a GI strain. We further reveal that the N-terminal 179-aa region of GII-NTPase is sufficient to mediate (i) vesicle formation, (ii) ER colocalization, (iii) the interaction with two other nonstructural proteins, including Nterm and P22, (iv) the formation of homodimers or homo-oligomers, and (v) the induction of cell apoptosis. Taken together, our findings emphasize that the virus-encoded NTPase must have multiple activities during viral replication or pathogenesis; however, these activities may vary somewhat among different genogroups.

## INTRODUCTION

Noroviruses are members of the family Caliciviridae and contain a positive-sense single-stranded RNA genome (1). Noroviruses can be classified into seven genogroups, designated genotype I (GI), GII, GIII, GIV, GV, GVI, and GVII, based on their genome sequence similarity (1, 2). Among them, viruses from GI, GII, and, occasionally, GIV can infect humans and cause acute gastroenteritis (3). Viral strains in each genogroup can be further divided into distinct genotypes according to their nucleotide and amino acid sequence diversity. In particular, variants of the GII.4 genotype have been recognized as the most widespread strains that result in pandemic outbreaks since the mid-1990s (4–6).

Despite the fact that human noroviruses (HuNVs) are now the leading cause of acute nonbacterial gastroenteritis (1, 7), multiple aspects regarding the mechanisms of viral genome replication or viral pathogenesis are still poorly understood. The poor understanding of HuNV replication and pathogenicity is due to the lack of an efficient and reliable cell culture system or an animal system for multicycle replication of HuNV. Although progress has been made in recent years regarding the identification of human permissive target cells for HuNV infection *in vitro* (8, 9) and the use of a plasmid-based replicon for HuNV propagation (10, 11), these newly established model systems have not yet been combined for investigations of the molecular mechanisms associated with virus-host interactions. Currently, most of our knowledge about HuNV biology is derived mainly from studies with animal caliciviruses such as murine norovirus (MNV; a strain of GV noroviruses).

The RNA genome of HuNV is about 7.6 kb in length and consists of three conserved open reading frames (ORF), including ORF1, ORF2, and ORF3 (12). Like all RNA genomes of caliciviruses, the 5′ end of HuNV genomic RNA is covalently linked to a virus-encoded protein known as VPg (viral protein, genome linked), and the 3′ end contains a poly(A) tail (1, 12). ORF1 in the viral genome encodes a large nonstructural polyprotein that can be further proteolytically cleaved into six mature proteins, including Nterm (N-terminal nonstructural protein, or NS1-2), NTPase (or NS3), P22 (or NS4), VPg (or NS5), Pro (proteinase, or NS6), and RdRp (RNA-dependent RNA polymerase, or NS7). ORF2 and ORF3 encode the major capsid protein (VP1) and the minor capsid protein (VP2), respectively. According to an integrated model of noroviral genome replication, the incoming viral RNA directly acts as an mRNA for viral protein synthesis during infection (13, 14). Following the synthesis of the ORF1 polyprotein precursor, Pro is responsible for the cleavage of the polyprotein into six mature nonstructural proteins. These viral nonstructural proteins may subsequently participate in the assembly of a replication complex where RdRp functions to catalyze the synthesis of the negative-sense RNA intermediate, genomic, and subgenomic RNA in cells. Although the principle functions of VPg, Pro, and RdRp in viral replication have been well documented, the exact roles of the other nonstructural proteins, including Nterm, NTPase, and P22, in cells still need further investigation.

For MNV or Norwalk virus (the prototype of GI noroviruses), Nterm and P22 previously were implicated in the remodeling of cell membranes, which may facilitate the assembly of the replication complex in the host cell (14–18). The NTPase protein of noroviruses is known to share sequence similarity with picornavirus 2C protein and the helicase of superfamily 3 (SF3) (19). However, biochemical characterization of the NTPase protein encoded by Southampton virus (SHV; a strain of GI noroviruses) revealed that the viral NTPase has both NTP-binding and NTP hydrolysis activities but lacks a helicase activity (19). More recently, Cotton et al. showed that when expressed individually, NTPase derived from MNV or Norwalk virus could induce the formation of vesicular structures in cells (20). Although they did not find colocalization of these vesicular structures with any particular organelle-specific markers, their studies suggested that the viral NTPase was dynamically associated with lipid membranes and microtubules. Despite the fact that the potential functions of these norovirus-encoded nonstructural proteins have been increasingly investigated, most of the published studies focused only on MNV or GI noroviruses (14). To date, very little information is available about the subcellular localization and function of the GII nonstructural proteins in cells.

In this report, we showed that the GII.4 nonstructural proteins expressed individually in cells displayed somewhat different intracellular localization patterns from those of the previously reported counterparts of GI and GV noroviruses (11, 20–23). We then performed a detailed characterization of the GII.4 NTPase, referred to here as GII-NTPase. Confocal immunofluorescence analysis revealed that GII-NTPase could form vesicular or nonvesicular structures in the cytoplasm, and the nonvesicular fraction of GII-NTPase showed a significant colocalization with the endoplasmic reticulum (ER) or mitochondria. The critical regions of GII-NTPase required for vesicle formation, ER colocalization, and mitochondrial colocalization were mapped in this study. Additionally, GII-NTPase could form homodimers/oligomers and physically interact with Nterm and P22 in the cell. Functional analysis revealed that GII-NTPase had a proapoptotic activity, which could be further enhanced by coexpression with Nterm or P22. Therefore, our findings strongly suggest that GII-NTPase is a multifunctional protein in cells.

## RESULTS

### Expression and subcellular localization of the GII.4 nonstructural proteins.

**FIG 1:**
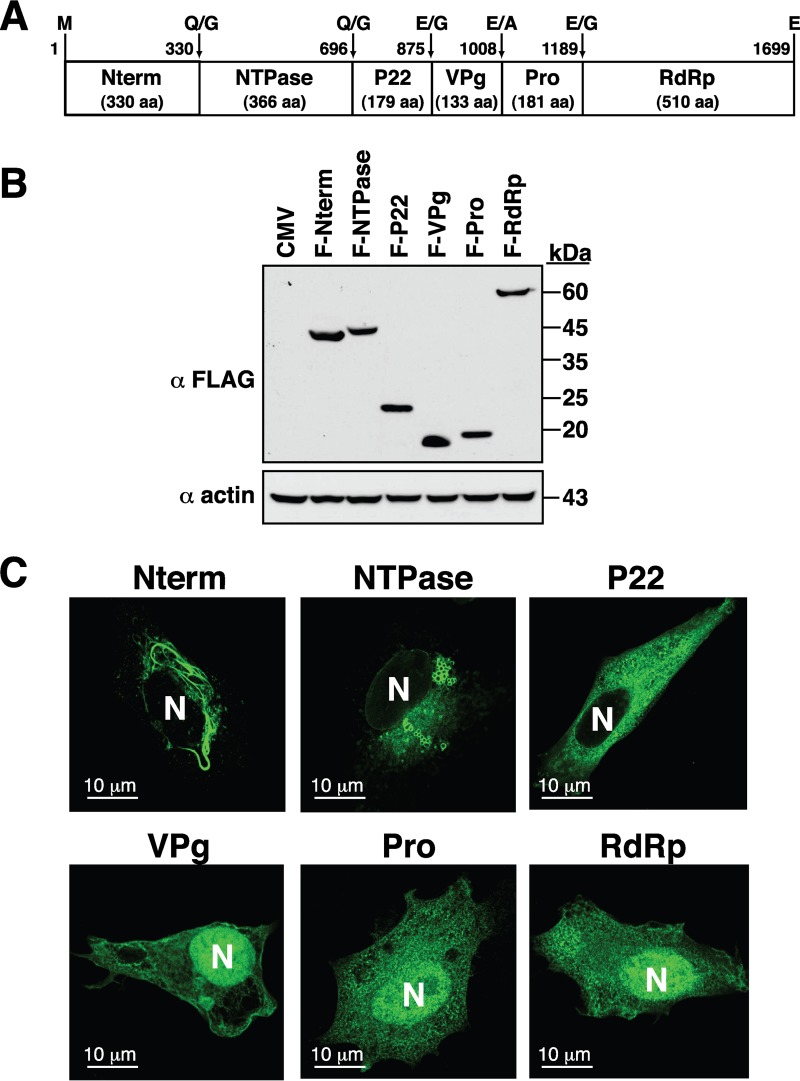
Expression and subcellular localization of six nonstructural proteins from a GII.4 HuNV strain. (A) Diagram of the six nonstructural proteins, including Nterm, NTPase, P22, VPg, Pro, and RdRp, within norovirus ORF1. The positions of proteolytic cleavage sites in the polyprotein precursor are indicated at the top of the diagram. (B) Western blot analysis of the viral nonstructural proteins expressed individually in 293T cells. The N-terminal FLAG-tagging expression plasmids that encode Nterm, NTPase, P22, VPg, Pro, or RdRp were transfected into 293T cells, and their protein expression was analyzed by immunoblotting using anti-FLAG antibody. CMV, cytomegalovirus. (C) Representative confocal microscopic images showing the subcellular localization of each nonstructural protein in A7 cells. Transfected cells were visualized using indirect immunofluorescence with anti-FLAG antibody. N, nucleus. Scale bars, 10 μm.

To determine the subcellular localization of the GII.4 nonstructural proteins, a full-length cDNA clone of a GII.4 HuNV strain, HuNV/GII.4/YJB1/2009/Chiayi, isolated from Chang-Gung Memorial Hospital at Chiayi (Taiwan), was used as the starting material for the DNA cloning. The cDNA fragments corresponding to the coding regions of Nterm, NTPase, P22, VPg, Pro, and RdRp were inserted into an N-terminal FLAG-tagging expression vector. The resultant expression plasmids were designated pCMV-F-Nterm, pCMV-F-NTPase, pCMV-F-P22, pCMV-F-VPg, pCMV-F-Pro, and pCMV-F-RdRp. When these expression plasmids were individually transfected into 293T cells, all of these nonstructural proteins were expressed normally in 293T cells and displayed predicted molecular masses on SDS-PAGE (Fig. 1A and B). The detected molecular masses of F-Nterm, F-NTPase, F-P22, F-VPg, F-Pro, and F-RdRp were 42, 45, 22, 18, 20, and 60 kDa, respectively. We subsequently analyzed their intracellular localization in human melanoma A7 cells using confocal microscopy (Fig. 1C). In confocal immunofluorescence analysis, we found that F-Nterm, F-NTPase, and F-P22 were exclusively localized in the cytoplasm, whereas F-VPg, F-Pro, and F-RdRp were distributed in both the cytoplasm and the nucleus (Fig. 1C). It is worth noting that F-Nterm consistently produced thread-like structures in the cell cytoplasm, whereas F-NTPase could form circular vesicular structures (Fig. 1C). In this study, we focused our attention on the analysis of the GII.4 norovirus-encoded NTPase, designated GII-NTPase, which consists of 366 amino acids (aa).

### The nonvesicular fraction of GII-NTPase significantly localizes to ER or mitochondria.

**FIG 2:**
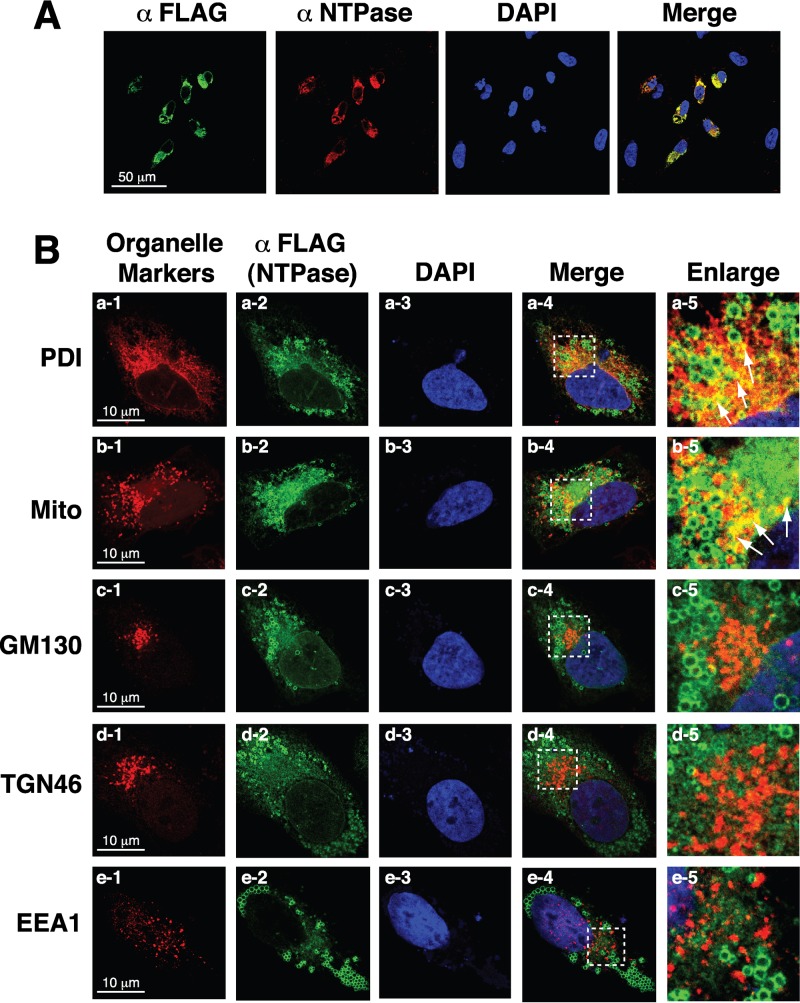
Nonvesicular fraction of GII-NTPase significantly localizes to the ER or mitochondria in cells. (A) The expression plasmid encoding F-NTPase was transfected into A7 cells. After 24 h of transfection, cells were fixed and dual labeled with an anti-FLAG antibody (green) and a rabbit polyclonal anti-NTPase antibody (red). Colocalization of signals recognized by the anti-FLAG and the anti-NTPase antibodies was detected as yellow color. Scale bar, 50 μm. (B) A7 cells expressing F-NTPase were fixed and dual stained with an anti-FLAG antibody (green) and a set of antibodies or a fluorescent dye (MitoTracker [Mito]) specific for organelle markers (red). Colocalization of F-NTPase with the signals of organelle-specific markers are shown in yellow (arrows). The dashed boxes in each merged image were enlarged and are shown at the right. As noted, the nonvesicular areas of F-NTPase colocalize with the signals of PDI (an ER marker) or Mito (a mitochondrial probe) but not those of GM130 (a *cis*-Golgi marker), TGN46 (a *trans*-Golgi marker), and EEA1 (an endosomal marker). Scale bars, 10 μm.

Before we further characterized the subcellular localization and biological functions of GII-NTPase, we initially investigated whether adding a FLAG tag to the N terminus of GII-NTPase interfered with the native structure of the protein. Therefore, an anti-NTPase antibody was generated using bacterially expressed GII-NTPase (aa 49 to 366) as an immunogen and was used in immunofluorescence staining experiments. In the experiments, A7 cells that were transfected with pCMV-F-NTPase were dually labeled with the anti-NTPase and anti-FLAG antibodies (Fig. 2A). As shown in Fig. 2A, full colocalization of the anti-FLAG and anti-NTPase signals was observed in transfected A7 cells by confocal microscopy, suggesting that the addition of a FLAG tag to GII-NTPase does not significantly affect the protein structure.

**FIG 3:**
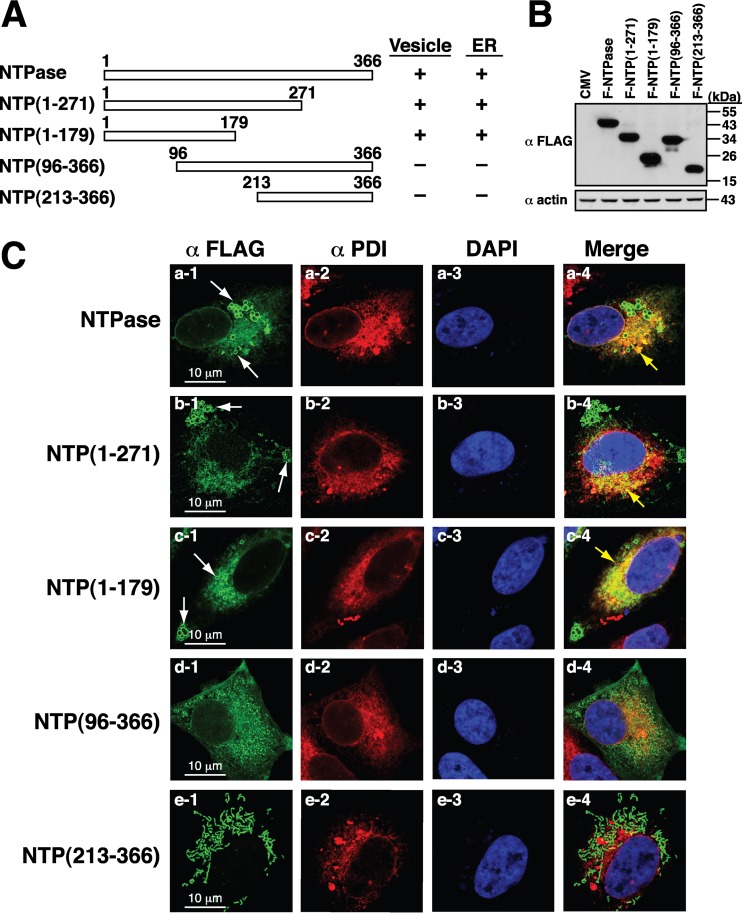
N-terminal 179-aa region of GII-NTPase is required for vesicle formation and ER colocalization. (A) Schematic diagram of the full-length and deletion constructs of F-NTPase. The abilities of F-NTPase and its deletions to promote vesicle formation and ER colocalization are summarized in the diagram. (B) Western blot analysis of F-NTPase and its deletions expressed in 293T cells. (C) Representative confocal immunofluorescence images of F-NTPase constructs (green) and PDI (red) in A7 cells. White arrows indicate the positions of vesicle-like structures formed by F-NTPase or its deletions. Yellow arrows represent the colocalization between the indicated F-NTPase constructs and PDI in the merged images. Scale bars, 10 μm.

**FIG 4:**
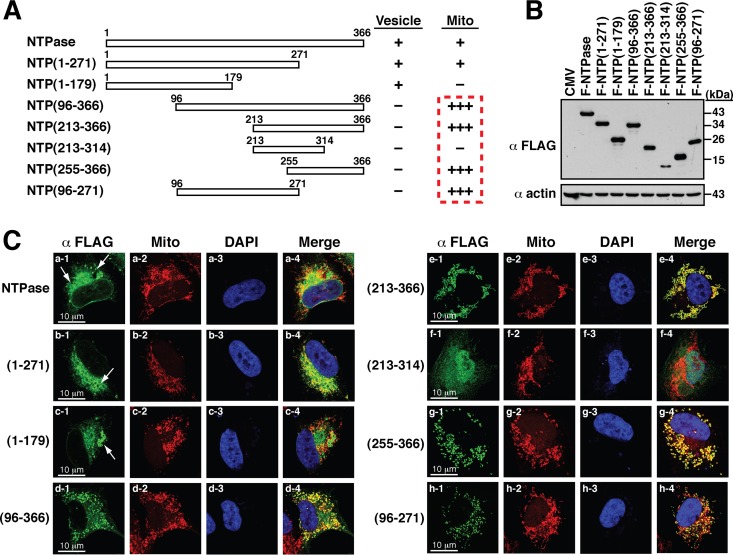
C-terminal region of GII-NTPase is involved in mitochondrial targeting. (A) Diagram of the full length and deletion constructs of F-NTPase. The abilities of various F-NTPase constructs to form vesicles or to localize to mitochondria in the cell cytoplasm are summarized. The degrees of mitochondrial targeting of these F-NTPase constructs are indicated by a plus sign. (B) Western blot analysis of the indicated F-NTPase constructs expressed in 293T cells. (C) Representative confocal microscopic images showing the localization of various F-NTPase constructs and MitoTracker signals in A7 cells. White arrows indicate vesicular induction by F-NTPase or its deletion mutants in the cell. It should be noted that several N-terminal deletion constructs, including NTP(96–366), NTP(213–366), NTP(255–366), and NTP(96–271), showed high degrees of colocalization with the signals of MitoTracker. Scale bars, 10 μm.

**FIG 5:**
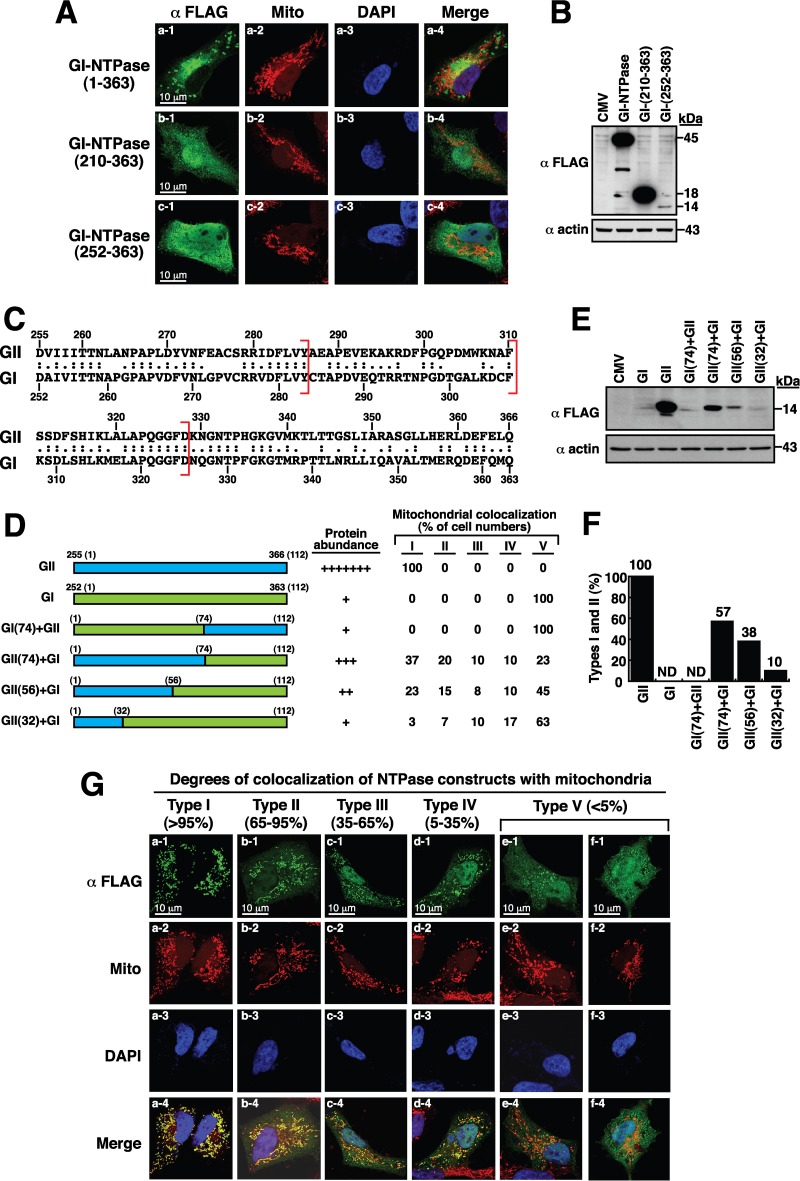
Full-length and N-terminal-deleted NTPase constructs from a GI norovirus strain do not show significant colocalization with mitochondria. (A) Immunofluorescence analysis of the subcellular localization of the full-length and N-terminal-deleted GI-NTPase constructs in A7 cells. As noted, GI-NTPase(1–363), GI-NTPase(210–363), and GI-NTPase(252–363) did not show substantial colocalization with mitochondria. Scale bars, 10 μm. (B) Western blot analysis of the full length and deletion constructs of GI-NTPase expressed in 293T cells. (C) Sequence alignment of amino acid residues between GII-NTPase from aa 255 to 366 and GI-NTPase from aa 252 to 363. (D) Diagram of domain-swapped NTPase constructs and summary of their protein abundance and mitochondrion-targeting ability. (E) The degrees of protein abundance for each NTPase chimeric construct, determined by Western blotting, are indicated by a plus sign. As noted, several domain-swapped NTPase constructs produced diversified phenotypes with various degrees of mitochondrial colocalization in transfected cells. (G) The heterogeneous cell populations were divided into five types (types I to V) according to the degrees of NTPase chimeric constructs colocalized with the mitochondria. Cells with the type I, II, III, IV, and V patterns were defined as >95%, 65 to 95%, 35 to 65%, 5 to 35%, and <5% portions, respectively, of NTPase constructs colocalized with the mitochondria. Scale bars, 10 μm. (F) The percentages of the type I and type II patterns in transfected cells for the indicated NTPase constructs were evaluated by counting at least 300 FLAG-positive cells (*n* > 300).

To further analyze the subcellular localization of GII-NTPase, A7 cells expressing F-NTPase were dual stained with an anti-FLAG antibody for F-NTPase and antibodies for organelle-specific markers, including protein disulfide isomerase (PDI; an ER marker), Golgi matrix protein of 130 kDa (GM130; a *cis*-Golgi marker), *trans*-Golgi network protein of 46 kDa (TGN46; a *trans*-Golgi marker), and early endosome antigen 1 (EEA1; an endosome marker). Additionally, MitoTracker (Mito), a mitochondrial labeling dye, was used to stain mitochondria in the cell. Confocal microscopic analysis revealed that F-NTPase substantially colocalized with the signals of PDI (Fig. 2B, row a) or MitoTracker (Fig. 2B, row b) but not with those of GM130, TGN46, and EEA1 (Fig. 2B, rows c to e). It should be noted, however, that the vesicle structure formed by F-NTPase did not show any significant colocalization with PDI or MitoTracker (Fig. 2B, a-5 and b-5).

### Mapping the critical regions of GII-NTPase involved in vesicle formation, ER colocalization, and mitochondrial colocalization.

We next attempted to map the critical regions of GII-NTPase essential for vesicle formation and for the colocalization of ER or mitochondria. Since little is known about the structure and function of GII-NTPase, a series of the N-terminal or C-terminal deletion constructs of F-NTPase were generated (Fig. 3A and B). At the start, the expression plasmids encoding F-NTPase deletion constructs were transfected into A7 cells, and the transfected cells were dual stained with anti-FLAG and anti-PDI antibodies (Fig. 3C). Confocal microscopic analysis showed that the C-terminal-deleted mutants, including F-NTPase(1–271) and F-NTPase(1–179), still retained the ability to form vesicles and colocalize with PDI (Fig. 3C, rows a to c). However, the F-NTPase deletions lacking the N-terminal region, such as F-NTPase(96–366) and F-NTPase(213–366), completely lost their ability to form vesicles and to colocalize with PDI (Fig. 3C, rows d and e). These results suggested that the N-terminal 179-aa region of GII-NTPase is important for vesicle formation and ER colocalization in the cell.

When the transfected cells were dual stained with anti-FLAG antibody and MitoTracker, we found that full-length F-NTPase and F-NTPase(1–271) showed a similar extent of mitochondrial colocalization (Fig. 4A and C, rows a and b). In contrast, further C-terminal deletion to aa 179, F-NTPase(1–179), completely abolished its localization to mitochondria (Fig. 4C, row c). Surprisingly, we found that two N-terminal deletion constructs, including F-NTPase(96–366) and F-NTPase(213–366), in the cell colocalized perfectly with MitoTracker signals (Fig. 4C, rows d and e). These results were unexpected and prompted us to generate two more deletion constructs, F-NTPase(255–366) and F-NTPase(213–314) (Fig. 4A and B). Confocal microscopy analysis showed that F-NTPase(255–366) still perfectly colocalized with mitochondria (Fig. 4C, row g). However, F-NTPase(213–314) lost its ability to localize to mitochondria (Fig. 4C, row f). Based on the results described above, there are at least three important implications in the localization of GII-NTPase to mitochondria. First, the C-terminal region from aa 255 to 366 of GII-NTPase, GII-NTPase(255–366), could be a potent mitochondrion-targeting domain. Second, the C-terminal region from aa 314 to the end might be critically involved in mitochondrial colocalization of GII-NTPase. Third, the N-terminal region of GII-NTPase might have a suppressive activity in inhibiting the mitochondrial targeting of GII-NTPase.

Besides the extreme C-terminal mitochondrion-targeting domain in GII-NTPase, we noticed that the mutant F-NTPase(1–271) partially localized to mitochondria to an extent comparable to that of full-length F-NTPase (Fig. 4C, compare rows a and b). The question therefore was raised as to whether F-NTPase(1–271) contained another mitochondrion-targeting domain. Since the N-terminal region might have an inhibitory activity toward the mitochondrial targeting of F-NTPase, we deleted the N-terminal 95-aa region from F-NTPase(1–271). Consistent with our hypothesis, high degrees of colocalization between F-NTPase(96–271) and mitochondria were detected in confocal microscopic analysis (Fig. 4C, row h). Taken together, our findings proposed that GII-NTPase has two separate mitochondrion-targeting domains.

### Difference in the mitochondrial localization between GI-NTPase and GII-NTPase.

As described above, GII-NTPase could localize to mitochondria in cells. We next investigated whether the NTPase protein derived from a GI norovirus strain, GI-NTPase, could also localize to mitochondria. The full-length GI-NTPase protein contains 363 amino acids and shares 52% identity (or 67% similarity) with GII-NTPase. When the GI-NTPase protein was expressed in A7 cells, vesicle-like structures induced by GI-NTPase could be observed in the cell; however, very limited colocalization between GI-NTPase and the signals of MitoTracker was detected (Fig. 5A, row a). Since GII-NTPase deletion constructs that lacks the N-terminal region, such as GII-NTPase(213–366) and GII-NTPase(255–366), gained the mitochondrial targeting function, the corresponding GI-NTPase deletions, including GI-NTPase(210–363) and GI-NTPase(252–363), also were generated (Fig. 5A and B). Unlike the GII-NTPase deletion mutants, GI-NTPase(210–363) and GI-NTPase(252–363) displayed diffused distribution in both the nucleus and the cytoplasm and did not show any significant colocalization with mitochondria (Fig. 5A, rows b and c). During the course of the experiments, we additionally found that GI-NTPase(252–363) was expressed at a much lower level than the full-length GI-NTPase and GI-NTPase(210–363) (Fig. 5B). Currently, we do not know why GI-NTPase(252–363) was expressed at very low levels in transfected cells. It is possible that the specific deletion of GI-NTPase from aa 210 to 252 causes a decrease in mRNA stability, mRNA translation efficiency, or protein stability.

An alignment of protein sequences between GII-NTPase(255–366) and GI-NTPase(252–363) revealed that both proteins share 53% amino acid identity (or 66% amino acid similarity) (Fig. 5C). To further investigate the difference in the localization between GI-NTPase(252–363) and GII-NTPase(255–366), reciprocal domain-swapping experiments were performed. We first constructed the mutants by replacing the N-terminal 74-aa region of GII-NTPase(255–366) with the corresponding N-terminal region of GI-NTPase(252–363) and vice versa (Fig. 5D). Two domain-swapped mutants, GI (74)+GII and GII (74)+GI, were generated (Fig. 5D). Confocal microscopic examination revealed that GI (74)+GII did not show any substantial colocalization with mitochondria (Fig. 5D and F). Interestingly, the GII (74)+GI chimeric construct in transfected cells exhibited a heterogeneous spectrum of its localization to mitochondria. Based on the degrees of mitochondrial colocalization, the GII (74)+GI-transfected cells could be divided into five types (types I to V) that showed >95%, 65 to 95%, 35 to 65%, 5 to 35%, and <5% of GII (74)+GI colocalized with the mitochondria, respectively (representative images are in Fig. 5G). It is worth noting that the proportions of GII (74)+GI-transfected cells with the type I, II, III, IV, and V patterns were found to be 37%, 20%, 10%, 10%, and 23%, respectively (Fig. 5D). The nonuniform patterns of the mitochondrial colocalization for the GII (74)+GI chimeric mutant implicate that intrinsic differences in cultured cells (e.g., mitochondrial dynamics and mitochondrial biogenesis) affect its mitochondrial targeting.

As shown above, the N-terminal 74-aa region of GII-NTPase(255–366) was demonstrated to be critical for supporting mitochondrial targeting. We generated another two domain-swapped mutants, GII (56)+GI and GII (32)+GI, which encompass the N-terminal 56 and 32 residues of GII-NTPase(255–366), respectively. Like the GII (74)+GI construct, both GII (56)+GI and GII (32)+GI also exhibited a wide spectrum of localization to mitochondria in transfected cells (Fig. 5D and G). When the mitochondrion-targeting ability of the NTPase chimeric mutants was evaluated, we found that the colocalization between these chimeric constructs and mitochondria was gradually reduced along with the shortening of the N-terminal region of GII-NTPase(255–366) in domain-swapped mutants (Fig. 5D and F). The percentages of the type I and type II patterns (corresponding to high degrees of colocalization with the mitochondria) for GII-NTPase(255–366), GII (74)+GI, GII (56)+GI, and GII (32)+GI in transfected cells were found to be 100%, 57%, 38%, and 10%, respectively (Fig. 5F). Since no specific mitochondrial targeting signal could be identified in GII-NTPase(255–366) using the domain-swapping approach, our results suggested that the targeting of these NTPase chimeric constructs to the mitochondria is dependent upon their protein structure but not a contiguous linear sequence motif.

### Interaction of GII-NTPase with itself and other nonstructural proteins.

**FIG 6:**
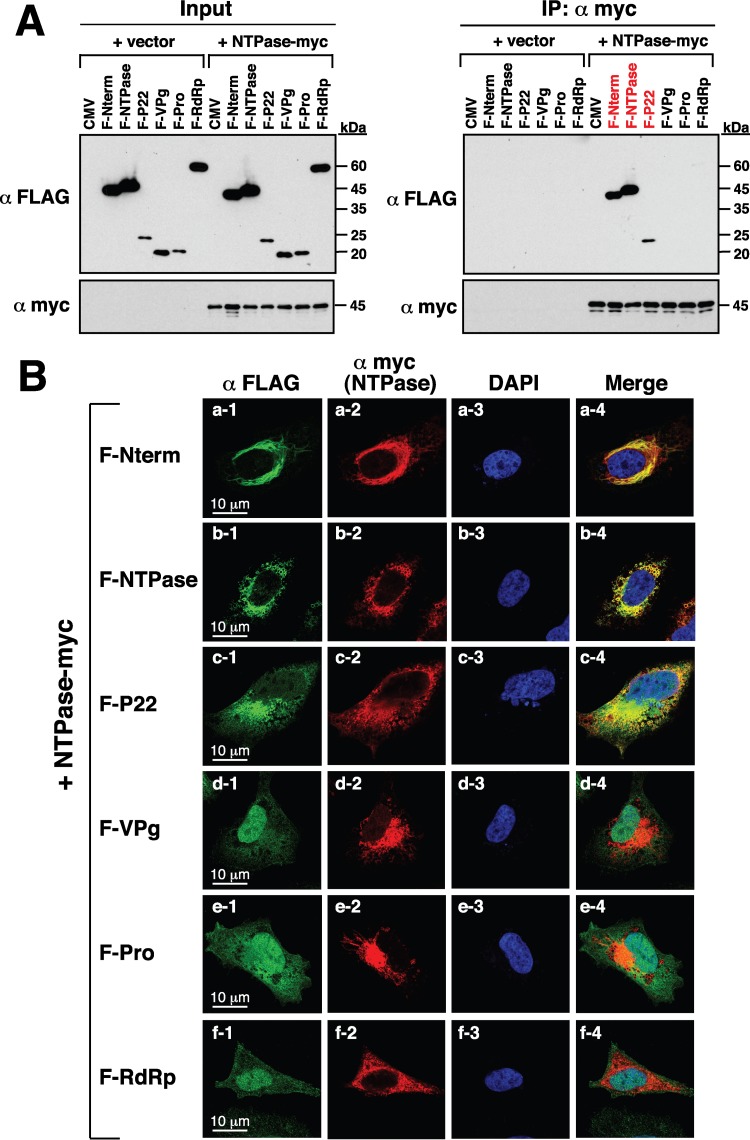
GII-NTPase physically interacts with itself as well as Nterm and P22 but not VPg, Pro, and RdRp. (A) Coimmunoprecipitation of GII-NTPase with each nonstructural protein derived from a GII.4 HuNV strain. 293T cells were cotransfected with an expression plasmid encoding myc-tagged GII-NTPase(NTPase-myc) and the plasmids expressing FLAG-tagged viral proteins. The cell lysates were prepared and immunoprecipitated using anti-myc antibody. The resultant immunoprecipitates were subsequently analyzed for the presence of FLAG-tagged viral proteins by immunoblotting. Input indicates the total cell lysate used in the immunoprecipitation (IP). (B) Confocal immunofluorescence images showing the colocalization of NTPase-myc with F-Nterm, F-NTPase, and F-P22 but not F-VPg, F-Pro, and F-RdRp. Double staining with an anti-myc antibody for NTPase-myc (red) and an anti-FLAG-antibody for FLAG-tagged viral proteins (green) were performed in the assay. Scale bars, 10 μm.

Due to the importance of the replication complex assembly during noroviral infection, we determined whether GII-NTPase physically interacts with itself or other nonstructural proteins. In the experiments, 293T cells first were cotransfected with the expression plasmid encoding a C-terminally myc-tagged NTPase(NTPase-myc) and the expression plasmid encoding F-Nterm, F-NTPase, F-P22, F-VPg, F-Pro, or F-RdRp (Fig. 6). Cell lysates of the transfected samples were immunoprecipitated using anti-myc antibody, and then the resultant immunoprecipitates were analyzed by Western blotting using anti-FLAG and anti-myc antibodies. We found that F-Nterm, F-NTPase, and F-P22, but not F-VPg, F-Pro, and F-RdRp, could be coimmunoprecipitated with NTPase-myc in the experiments (Fig. 6A, right). In confocal microscopic analysis, we also demonstrated that NTPase-myc specifically colocalized with F-Nterm, F-P22, and F-NTPase but not F-VPg, F-Pro, and F-RdRp in transfected A7 cells (Fig. 6B).

### Mapping the interaction region of GII-NTPase with Nterm and P22.

**FIG 7:**
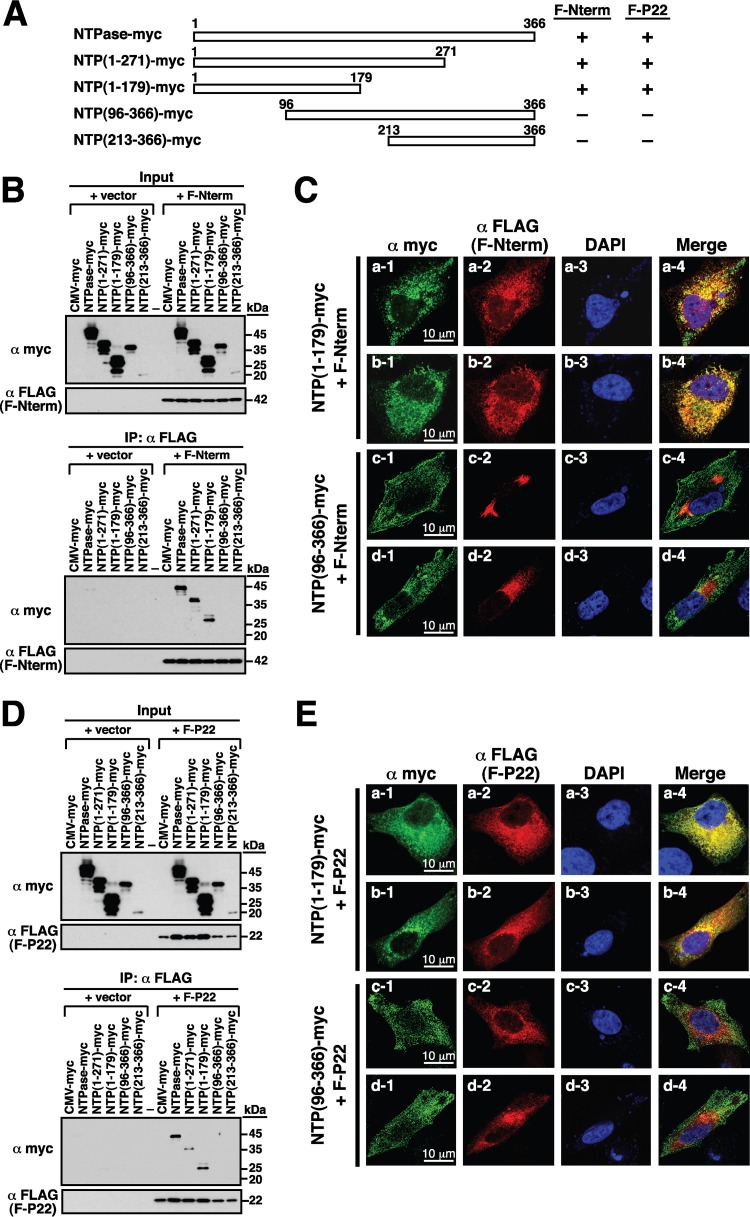
N-terminal 179-aa region of GII-NTPase is essential for the interaction with Nterm and P22. (A) Summary of the interaction of NTPase-myc deletion constructs with F-Nterm or F-P22. (B) Analysis of the interaction between different NTPase-myc deletions and F-Nterm by coimmunoprecipitation. The F-Nterm-expressing plasmid or an empty vector was cotransfected with the indicated expression plasmids encoding NTPase-myc or its deletions into 293T cells. Cell lysates were prepared and immunoprecipitated using anti-FLAG antibody, and then the immunoprecipitates were analyzed by Western blotting using anti-myc antibody. (C) Confocal microscopic analysis of the interaction between F-Nterm (red) and NTPase(1–179)-myc or NTPase(96–366)-myc (green) in transfected A7 cells. Scale bars, 10 μm. (D) Coimmunoprecipitation analysis of F-P22 with different NTPase-myc deletions. 293T cells were cotransfected with an empty vector or the F-P22-expressing plasmid and the indicated plasmids encoding NTPase-myc or its deletions. After immunoprecipitation with anti-FLAG antibody, the immunoprecipitates were analyzed for the presence of NTPase-myc deletion constructs by immunoblotting using anti-myc antibody. (E) Confocal microscopic analysis of the interaction between F-P22 (red) and NTPase(1–179)-myc or NTPase(96–366)-myc (green) in transfected A7 cells. Scale bars, 10 μm.

To further map the critical region of NTPase responsible for the interaction with Nterm or P22, different deletion constructs of NTPase-myc (Fig. 7A) were cotransfected with the plasmid expressing F-Nterm or F-P22 into 293T cells. Immunoprecipitation was carried out using anti-FLAG antibody, and then the precipitated lysates were analyzed by Western blotting using anti-FLAG and anti-myc antibodies (Fig. 7B and D). Results from the Western blot analysis revealed that the full-length NTPase-myc, NTPase(1–271)-myc, and NTPase(1–179)-myc, but not NTPase(96–366)-myc and NTPase(213–366)-myc, could be coimmunoprecipitated with F-Nterm or F-P22 (Fig. 7B and D, bottom). During the course of the experiments, we noticed that the levels of NTPase(96–366)-myc and NTPase(213–366)-myc apparently were lower than those of other NTPase-myc deletion mutants in cell lysates (Fig. 7B and D, top). The reduced levels of these two mutant proteins in the immunoprecipitation experiments were not due to their lower protein expression in cells. In fact, when the same transfected cells were lysed with Laemmli sample buffer, we found that all mutant proteins were expressed equally (data not shown), indicating that NTPase(96–366)-myc and NTPase(213–366)-myc have reduced solubility in the immunoprecipitation assay buffer. In confocal microscopic analysis, we also demonstrated that NTPase(1–179)-myc, but not NTPase(96–366), was able to interact with F-Nterm and F-P22 in transfected A7 cells (Fig. 7C and E).

### Mapping of the critical regions for the GII-NTPase self-interaction.

**FIG 8:**
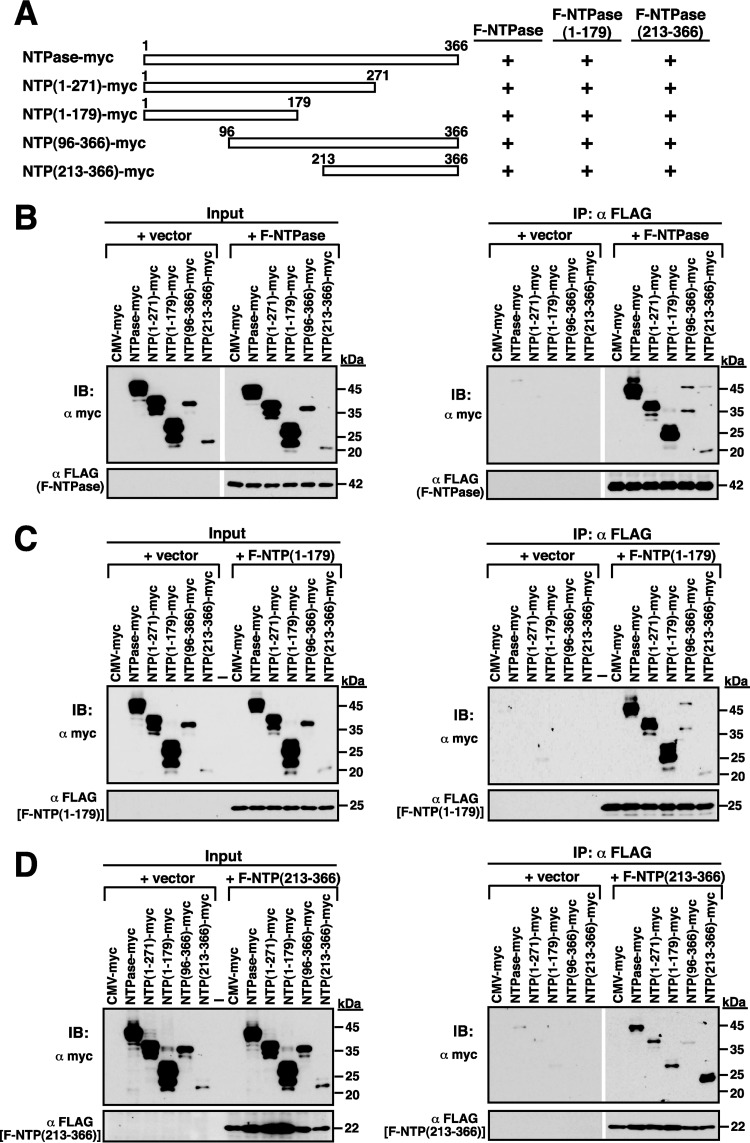
Mapping of the self-interacting domains in GII-NTPase. (A) Summary of the interactions among different domains of GII-NTPase. The myc- and FLAG-tagged protein constructs were used to determine the self-interaction of GII-NTPase. The interactions of F-NTPase(B), F-NTPase(1–179) (C), or F-NTPase(213–366) with myc-tagged NTPase deletion constructs were analyzed using the coimmunoprecipitation assay. After immunoprecipitation with anti-FLAG antibody, the resultant immunoprecipitates were analyzed for the presence of myc-tagged NTPase constructs by immunoblotting (IB) using anti-myc antibody.

**FIG 9:**
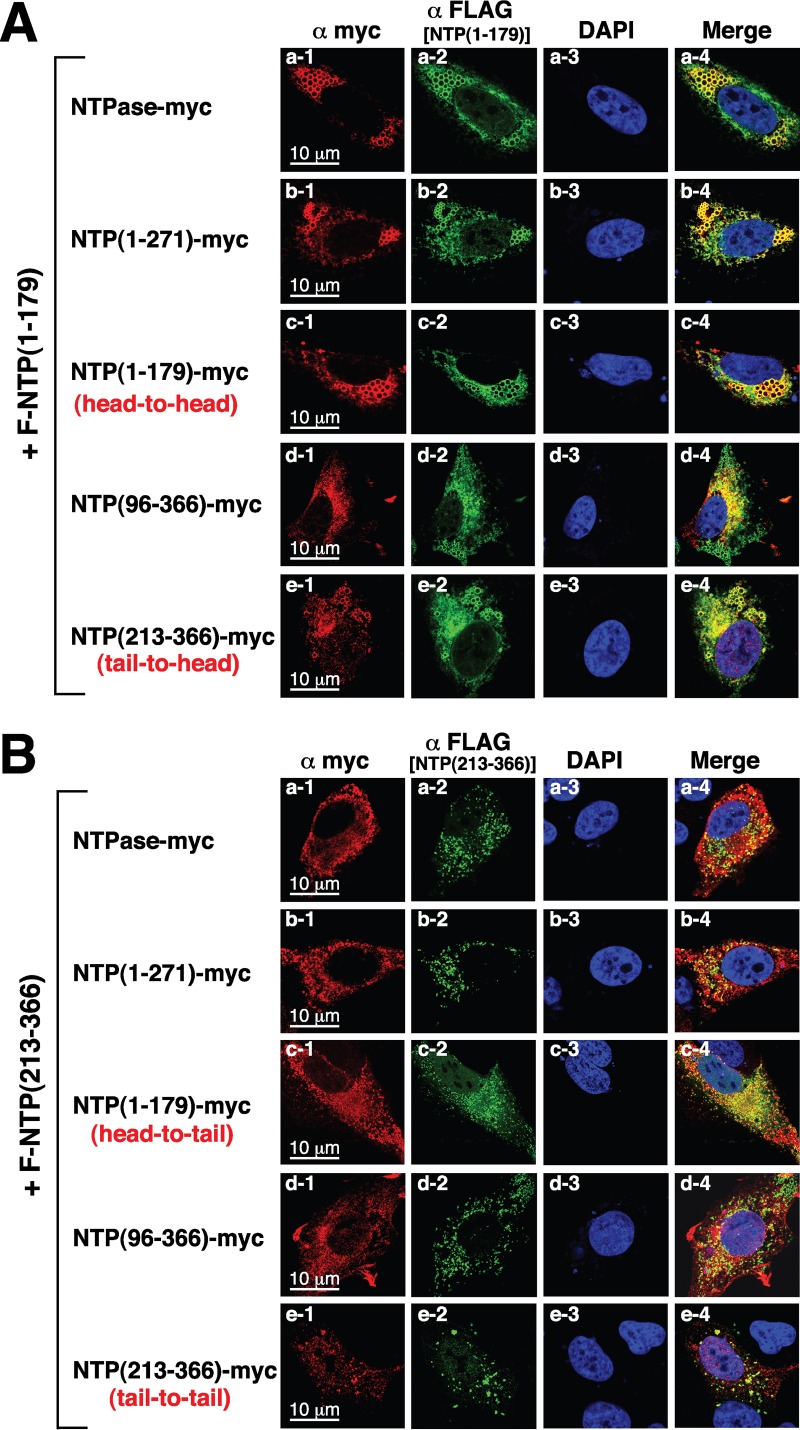
Analysis of a head-to-head, tail-to-tail, or head-to-tail interaction of GII-NTPase in transfected cells by confocal immunofluorescence microscopy. (A) A7 cells were cotransfected with an F-NTPase(1–179)-expressing plasmid and the plasmids expressing NTPase-myc or its deletions. The interaction between F-NTPase(1–179) (green) and the full length or deletion mutants of NTPase-myc (red) were determined in double-staining experiments. Scale bars, 10 μm. (B) An F-NTPase(213–366)-expressing plasmid was cotransfected with the plasmids expressing the full length or deletion constructs of NTPase-myc into A7 cells. The interaction of F-NTPase(213–366) with the full length or deletion mutants of NTPase-myc in A7 cells was characterized using confocal immunofluorescence microscopy. The head-to-head, tail-to-tail, or head-to-tail configuration of the GII-NTPase self-interaction also are pointed out in the images. Scale bars, 10 μm.

To further investigate the GII-NTPase self-interaction, coimmunoprecipitation analyses were performed using protein lysates of 293T cells that were cotransfected with the plasmid encoding F-NTPase and the plasmid encoding the full length or deletion constructs of NTPase-myc (Fig. 8A and B). After immunoprecipitation of F-NTPase using anti-FLAG antibody, we found that all tested NTPase-myc deletion proteins could be coimmunoprecipitated with F-NTPase, including NTPase(1–271)-myc, NTPase(1–179)-myc, NTPase(96–366)-myc, and NTPase(213–366)-myc (Fig. 8B, right). Among them, the mutants NTPase(1–179)-myc and NTPase(213–366)-myc are nonoverlapping constructs, suggesting that GII-NTPase contains at least two self-interaction domains. To determine whether the GII-NTPase self-interaction occurred in a head-to-head, tail-to-tail, or head-to-tail configuration, 293T cells first were cotransfected with an expression plasmid encoding F-NTPase(1–179) and the plasmid expressing the full length or deletion constructs of NTPase-myc (Fig. 8C). After immunoprecipitation with anti-FLAG antibody, Western blot analysis showed that all NTPase-myc deletion constructs tested also could be coimmunoprecipitated with F-NTPase(1–179) (Fig. 8C, right). We then determined the interaction between F-NTPase(213–366) and NTPase-myc deletion constructs by coimmunoprecipitation assay. Similarly, we also found that all NTPase-myc deletion constructs could be coimmunoprecipitated with F-NTPase(213–366) (Fig. 8D). These results strongly implied that GII-NTPase could form homodimers/oligomers through a head-to-head, head-to-tail, or tail-to-tail configuration. To further confirm our coimmunoprecipitation experiments, the interactions of F-NTPase(1–179) or F-NTPase(213–366) with different NTPase-myc deletion constructs in transfected A7 cells were examined by confocal microscopy (Fig. 9). Consistent with coimmunoprecipitation results, we found that F-NTPase(1–179) or F-NTPase(213–366) colocalized with full-length NTPase-myc, NTPase(1–271)-myc, NTPase(1–179)-myc, NTPase(96–366)-myc, and NTPase(213–366)-myc (Fig. 9A and B). In particular, F-NTPase(1–179) interacting with NTPase(1–79)-myc, a head-to-head configuration, gave rise to vesicular structures (Fig. 9A, row c), whereas F-NTPase(213–366) interacting with NTPase(213–366)-myc, a tail-to-tail configuration, formed punctate structures (Fig. 9B, row e). When both F-NTPase(1–179) and NTPase(213–366)-myc or both F-NTPase(213–366) and NTPase(1–179)-myc were coexpressed in A7 cells (a head-to-tail configuration), vesicular and/or punctate structures could be observed in colocalized areas (Fig. 9A, row e, and B, row c).

### GII-NTPase possesses a proapoptotic activity that can be enhanced by coexpression of Nterm or P22.

**FIG 10:**
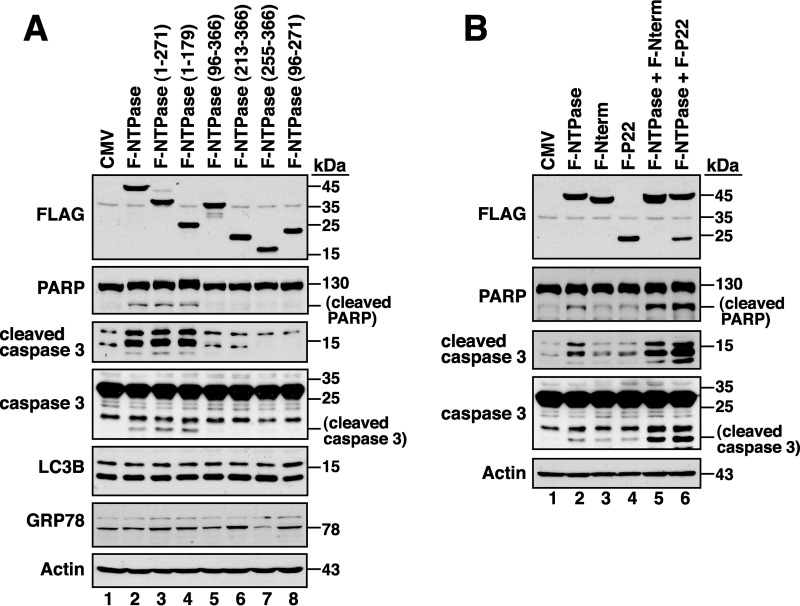
GII-NTPase possesses proapoptotic activity, which can be further enhanced by Nterm or P22. (A) Effects of GII-NTPase or its deletion mutants on cell apoptosis, autophagy, and ER stress. After transfection of the indicated GII-NTPase expression plasmids into 293T cells for 24 h, the expression of specific markers for apoptosis (PARP or caspase 3), autophagy (LC3B), and ER stress (GRP78) were examined by Western blotting. (B) Enhancement of the GII-NTPase-mediated apoptosis by Nterm or P22. 293T cells were cotransfected with the indicated plasmids expressing GII-NTPase, Nterm, or P22. After 24 h of transfection, the expression of cleaved PARP and caspase 3 in the cells was analyzed by Western blotting.

Since GII-NTPase could localize to mitochondria, ER, or other unknown areas in the cytoplasm, we examined whether GII-NTPase triggered cellular stress responses such as apoptosis, unfolded protein response (UPR), or autophagy. The expression of specific markers for apoptosis (cleavage of PARP and caspase 3), UPR (GRP78 upregulation), and autophagy (LC3B-II upregulation) therefore were evaluated in 293T cells transfected with empty vector or GII-NTPase-expressing plasmid. Compared to the control cells, cells expressing GII-NTPase substantially induced the expressions of cleaved PARP and cleaved caspase 3 (Fig. 10A, lane 2). However, GII-NTPase expression did not affect the overall level of GRP78 and LC3B-II in the cell (Fig. 10A, lane 2). When different GII-NTPase deletion constructs were expressed individually in 293T cells, we found that F-NTPase(1–271) and F-NTPase(1–179) were sufficient to promote the cleavage of PARP and caspase 3 (Fig. 10A, lanes 3 and 4). However, the deletion constructs that had a potent mitochondrion-targeting ability, including F-NTPase(96–366), F-NTPase(213–366), F-NTPase(255–366), and F-NTPase(96–271), did not induce the cleavage of PARP and caspase 3 (Fig. 10A, lanes 5 to 8). These results indicated that GII-NTPase contains proapoptotic activity; however, the proapoptotic activity is independent of its mitochondrial targeting.

Since Nterm or P22 was capable of interacting with GII-NTPase as described above, we then investigated whether Nterm or P22 modulated the proapoptotic activity of GII-NTPase. Compared to the control cells, cells expressing Nterm or P22 alone did not significantly induce the cleavage of PARP and caspase 3 (Fig. 10B, lanes 2 and 3). However, coexpression of Nterm or P22 with GII-NTPase in cells substantially augmented the levels of cleaved PARP and caspase 3 (Fig. 10B, lanes 5 and 6).

## DISCUSSION

Although the genotype GII.4 strains of HuNV are the major agents of global gastroenteritis outbreaks in recent years, studies dealing with the biological functions of the GII.4 nonstructural proteins are seldom performed. Previous studies from GI or GV noroviruses have shown that all viral nonstructural proteins (including Nterm, NTPase, P22, VPg, Pro, and RdRp) were intimately associated with the viral replication complex in the cell cytoplasm during viral infections (21–25). However, here we showed that when expressed individually in cells, three GII.4 nonstructural viral proteins, including VPg, Pro, and RdRp, were distributed in both the cytoplasm and the nucleus (Fig. 1C). In fact, a GII.3 HuNV-based cell culture model has been previously established by Katayama et al. (10) using a full-length viral replicon DNA vector, and we noticed that RdRp, and probably also Pro, expressed from the GII.3-based replicon vector in COS7 cells was localized in both the cytoplasm and the nucleus (10). Although the main functions of the noroviral nonstructural proteins are for viral genome replication in the cytoplasm, the nuclear distribution of specific nonstructural viral proteins implies that they possess diverse biological actions in host cells. Actually, the nuclear localization of nonstructural viral proteins is not uncommon and has been described in a number of other related RNA virus families (26–29). In the case of picornaviruses, both 3C protease (equivalent to the noroviral Pro) and 3D polymerase (equivalent to the noroviral RdRp) are known to enter the host nucleus (26–29). Numerous studies have shown that the picornaviral 3C protease could cleave cellular transcriptional regulators or polyadenylation processing factors in the nucleus to modulate host gene expression (26–28), whereas the picornaviral 3D polymerase could target the pre-mRNA processing factor 8 (Prp8) to prevent mRNA maturation (29). Thus, for future research, it would be interesting to understand whether the nuclear localization of the GII norovirus-encoded Pro or RdRp modulates specific host metabolic pathways. Although the Nterm, NTPase, and P22 proteins derived from GII.4 noroviruses were found exclusively in the cytoplasm, we would like to know whether these GII.4 viral proteins have the same intracellular localizations or functions as those of the previously reported counterparts derived from the GI and GV noroviruses. Here, we particularly focus our attention on characterizing the intracellular localization and the potential function of GII-NTPase.

### Subcellular localization of GII-NTPase in cells.

Previously, Cotton et al. (20) showed that the NTPase protein from MNV (a strain of GV noroviruses) or Norwalk virus (a strain of GI noroviruses) could induce the formation of vesicle-like structures in the cell cytoplasm. In agreement with their published data, we also found that GII-NTPase was capable of inducing the formation of vesicles in the cytoplasm. Currently, we do not know whether the circular vesicular structure is made up of only GII-NTPase or the combination of GII-NTPase with other cellular proteins. In addition to vesicular structures, a nonvesicular fraction of GII-NTPase was also detected diffusely within the cytoplasm. When specific organelle markers were costained with GII-NTPase, vesicle-like structures formed by GII-NTPase did not colocalize with any tested organelle-specific markers, including PDI, MitoTracker, GM130, TGN46, and EEA1 (Fig. 2B). These results are also consistent with the main findings reported by Cotton et al. (20) using GI and GV NTPase proteins. However, we showed here that the nonvesicular fraction of GII-NTPase substantially colocalized with the signals of PDI or MitoTracker (Fig. 2B). Deletion analysis of GII-NTPase revealed that the N-terminal 179-aa region is required for vesicle formation and for colocalization with PDI (Fig. 3), whereas the C-terminal region is responsible for the colocalization with MitoTracker (Fig. 4). Two functional mitochondrion-targeting domains in the C-terminal region of GII-NTPase were identified in the study and are located in the regions from aa 96 to 271 and from aa 255 to 366 of GII-NTPase (Fig. 4). Interestingly, although GII-NTPase contains two intrinsic mitochondrion-targeting domains, the full-length GII-NTPase is not predominantly localized to mitochondria (Fig. 2B, row b). These findings indicate that the N-terminal portion of GII-NTPase has an inhibitory action in its mitochondrial targeting. Due to the fact that GII-NTPase could interact with a variety of viral or cellular proteins during viral genome replication, we believe that different protein-protein interactions critically affect the targeting of GII-NTPase to mitochondria, ER, or other unknown areas. Further investigations may be required to determine whether and how viral or cellular proteins dynamically regulate the intracellular localization of GII-NTPase.

### Potential mechanisms and roles of GII-NTPase targeting to mitochondria.

Sequence analysis has revealed that the mitochondrion-targeting domains of GII-NTPase do not share significant homology with any known cellular or other viral proteins that are destined for mitochondria. Although GI-NTPase protein used in the study shares 52% amino acid identity with GII-NTPase and forms vesicle-like structures in the cytoplasm, the GI-NTPase protein did not produce substantial colocalization with mitochondria (Fig. 5A). This is consistent with the previous report that NTPase derived from Norwalk virus did not localize to mitochondria (20). In domain-swapping experiments using both GII-NTPase(255–366) and GI-NTPase(252–363), we showed that along with the shortening of the N-terminal part of GII-NTPase(255–366) in domain-swapped mutants, their mitochondrial targeting ability was also gradually reduced (Fig. 5D and F). These findings indicate that the N-terminal part of GII-NTPase(255–366) appears to be more important for the regulation of its mitochondrial targeting than the C-terminal part. However, we failed to identify a specific linear sequence motif within GII-NTPase(255–366) sufficient for the mitochondrial targeting using the domain-swapping approach. From these findings, it is possible that the targeting of GII-NTPase(255–366) to mitochondria is mediated through a specific protein structure rather than a short primary sequence motif. Additionally, it should be noted that most domain-swapped mutants presented in the study exhibited a wide spectrum of localization to mitochondria in transfected cells (Fig. 5D and G). The nonuniform patterns of mitochondrial colocalization for these domain-swapped mutants implied that intrinsic differences in cultured cells (e.g., mitochondrial fusion, fission, mitophagy, or biogenesis) influence their mitochondrial targeting.

Although the functional significance regarding the mitochondrial targeting of GII-NTPase in cells still remains obscure, three potential roles can be proposed. First, for many positive-sense RNA viruses, viral genome replication usually occurs in or on intracellular membranes. The association between GII-NTPase and mitochondria via the mitochondrion-targeting domains may expand the usage of intracellular membranes to facilitate cytoplasmic viral genome replication. Second, GII-NTPase is known to have NTP-binding and NTP-hydrolyzing activities (19), which may be involved in the modulation of central energy metabolism by ATP hydrolysis in cells. The association between GII-NTPase and mitochondria via the mitochondrion-targeting domains may increase the energy supply for viral replication. Third, it is well known that the maintenance of mitochondrial homeostasis is critical for cell survival under stress conditions. Thus, the association between GII-NTPase and mitochondria via the mitochondrion-targeting domains may be linked to prevention or promotion of cell apoptosis at specific stages during viral replication. To distinguish these possibilities, further investigations are needed. Moreover, it is important to note that the mitochondrion-targeting domains of GII-NTPase expressed in cells not only fully colocalized with mitochondria but also significantly affected mitochondrial morphology (Fig. 4C, rows d, e, g, and h). Unlike the normal cell that generally contains a heterogeneous pattern of mitochondrial morphology with the combination of small fragmented globules and tubular threads (30–32), the cells expressing GII-NTPase(96–366), GII-NTPase(213–366), GII-NTPase(255–366), or GII-NTPase(96–271) consistently exhibited a homogeneous pattern of mitochondrial morphology with either globules or tubular threads (Fig. 4, rows d, e, g, and h).

### Multiple functional activities for the N-terminal region of GII-NTPase.

**FIG 11:**
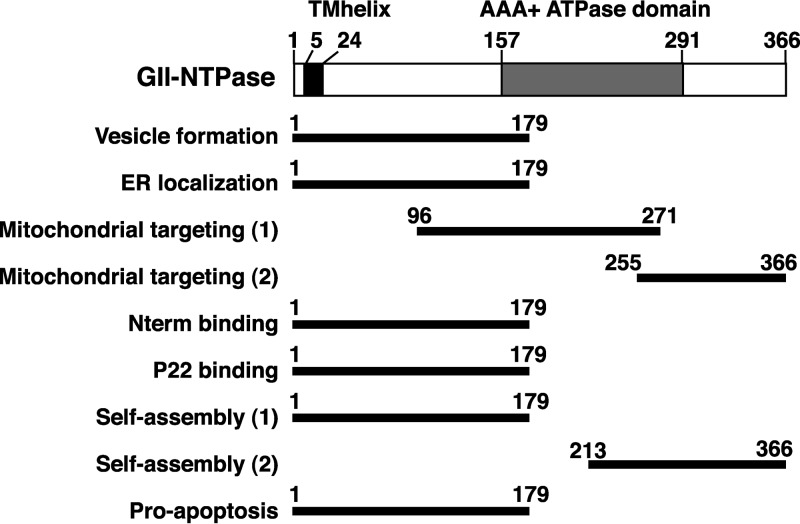
Predicted domain structure of GII-NTPase and summary of the identified regions essential for its specific functions. Numbers in the figure represent amino acid residues. The diagram at the top of the figure indicates a transmembrane helix (TMhelix; aa 5 to 24) and an ATPase domain of the AAA+ superfamily (aa 157 to 291), predicted via InterPro tools (http://www.ebi.ac.uk/interpro/) (33). The bottom of the figure represents the positions of different functional regions mapped in the study.

The full-length GII-NTPase contains 366 amino acids, in which a transmembrane helical motif located from aa 5 to 24 and an AAA+ ATPase domain located from aa 157 to 291 could be predicted via InterPro tools (http://www.ebi.ac.uk/interpro/) (33). Functional analysis of GII-NTPase in the study has revealed that the N-terminal 179-aa region may have multiple activities in cells (summarized in Fig. 11). Besides acting as an inhibitory region in mitochondrial targeting, the N-terminal 179-aa region is also necessary and sufficient for mediating vesicular formation (Fig. 3), ER colocalization (Fig. 3), the interaction with Nterm or P22 (Fig. 7), the assembly of homodimers or homo-oligomers (Fig. 8 and 9), and the induction of cell apoptosis (Fig. 10). Currently, we do not know whether the localization of GII-NTPase to the ER is mediated through a short ER-targeting sequence within the protein or through an interaction with other ER-associated cellular proteins. This issue will be addressed in future studies. Furthermore, the relationship between the diverse activities in the N-terminal region of GII-NTPase still remains to be determined. Due to the striking morphological differences between vesicular and nonvesicular (ER localization) structures formed by the N-terminal 179-aa region of GII-NTPase, the compositions of both vesicular and nonvesicular structures might be different in the cell. In this case, it may be interesting to know whether the formation of vesicular or ER-associated nonvesicular structures is linked to lipid droplets (LDs), the lipid-rich cytoplasmic organelles critical for lipid metabolism and energy homeostasis (34). Since LDs often vary in size in a given cell and can be divided into various types with different protein compositions (35), characterizing the association of GII-NTPase with specific LDs is under way. Additionally, although GII-NTPase could form homodimers or homo-oligomers through either a head-to-head, tail-to-tail, or head-to-tail configuration, the regularly shaped vesicles were only observed in a head-to-head configuration (Fig. 9A, row c). These findings indicate that vesicular formation is attributed to a higher-order self-interaction of the N-terminal region of GII-NTPase.

Previous studies from MNV have showed that MNV infection triggered apoptosis in RAW264.7 cells through the mitochondrial pathway, which was characterized by the activation of caspase 9 and caspase 3 as well as downregulation of survivin (36). Furthermore, Herod et al. (37) demonstrated that only expression of the MNV ORF1 polyprotein in HEK293 cells was sufficient to induce cell apoptosis. However, no single nonstructural protein of noroviruses is known to be associated with induction of apoptosis. We showed here that the expression of GII-NTPase alone is capable of promoting apoptosis (Fig. 10A), although the mechanism employed by GII-NTPase to induce apoptosis still remains unknown. Surprisingly, we found that the proapoptotic activity of GII-NTPase was independent of its localization to mitochondria (Fig. 10A). Although Nterm or P22 alone did not show significant activity on cell apoptosis, we did find that Nterm or P22 augmented the proapoptotic activity of GII-NTPase. These findings strongly suggest that cooperative interactions among different nonstructural proteins during viral infection critically modulate cellular metabolism, thereby affecting host cell death and survival.

In summary, we report the subcellular localization and various functions of GII-NTPase in the present study. Our findings emphasize that the NTPase protein encoded by noroviruses must have multiple activities during viral replication; however, these activities may vary somewhat among different genogroups.

## MATERIALS AND METHODS

### Cell cultures and transfections.

293T is a human embryonic kidney cell line that harbors the E1 region of adenovirus and the simian virus 40 T antigen (38). A7 is a melanoma cell line (39). 293T and A7 cells were cultured in high-glucose DMEM (Dulbecco's modified Eagle's medium) supplemented with 10% fetal bovine serum. Transfection experiments were performed using Lipofectamine 2000 (Invitrogen) according to the manufacturer's protocol. Briefly, 293T cells (8 × 10^5^) and A7 cells (1.5 × 10^5^) were grown in 6-well culture plates for 16 to 24 h and then transfected with 3 μg of plasmid DNA in 7.5 μl of Lipofectamine and 1 μg of plasmid DNA in 2.5 μl of Lipofectamine, respectively.

### Isolation of GI and GII norovirus strains.

Stool samples were collected from patients with acute gastroenteritis in Chang-Gung Memorial Hospital at Chiayi (Taiwan). The protocol was approved by the Institutional Review Board of Chang-Gung Memorial Hospital (IRB number 97-1761B). Viral RNAs were extracted from stool supernatants using a QIAamp viral RNA minikit according to the manufacturer's instructions (Qiagen, Germany). Subsequently, viral RNAs were converted into cDNA by reverse transcription, followed by DNA cloning and sequencing. Complete genome sequences of two norovirus strains corresponding to a GII.4 strain, HuNV/GII.4/YJB1/2009/Chiayi, and a GI strain, HuNV/GI/CYY1/2009/Chiayi, were used in the study. The GenBank accession numbers for the genome sequences of the GII.4 strain, HuNV/GII.4/YJB1/2009/Chiayi, and the GI strain, HuNV/GI/CYY1/2009/Chiayi, are MG049692 and MG049693, respectively. As noted, the amino acid sequence of GII-NTPase used here shows 99% amino acid identity (364 out of 366 residues) with most of the previously published sequences for different GII.4 variants. The GI-NTPase protein from the HuNV/GI/CYY1/2009/Chiayi strain is also highly homologous (82 to 99% amino acid identity) with those of the previously reported GI noroviruses.

### Plasmid construction.

The full-length cDNA clone of a GII.4 norovirus strain, HuNV/GII.4/YJB1/2009/Chiayi, was constructed by assembling different overlapping cDNA clones that were generated through reverse transcription. The full-length viral cDNA clone then was used as a template in PCR for the amplification of a target DNA region. To construct the expression plasmids encoding Nterm, NTPase, P22, VPg, Pro, and RdRp, the corresponding DNA fragments amplified by PCR were cloned into pFLAG-CMV-2 (Sigma). The resultant expression plasmids were designated pCMV-F-Nterm, pCMV-F-NTPase, pCMV-F-P22, pCMV-F-VPg, pCMV-F-Pro, and pCMV-F-RdRp. The expression plasmid pCMV-NTPase-myc was generated by inserting the NTPase coding region into pCMV-3Tag-4 (Stratagene), a C-terminal myc-tagging vector. Similarly, different expression plasmids encoding NTPase deletions were generated by insertion of PCR-amplified cDNA fragments into either pFLAG-CMV-2 or pCMV-3Tag-4. To construct the plasmids expressing the full length or deletion constructs of GI-NTPase, viral DNA fragments were obtained through PCR amplification using a cDNA clone of the GI norovirus strain (HuNV/GI/CYY1/2009/Chiayi) as a template. The domain-swapped mutants between GI-NTPase(252–363) and GII-NTPase(255–366) were constructed by using the overlapping PCR method (40).

### Western blot analysis.

Western blot analysis was carried out as described previously (41). Briefly, cells were lysed with Laemmli sample buffer (62.5 mM Tris-HCl [pH 6.8], 2% SDS, 10% glycerol, and 5% 2-mercaptoethanol) or immunoprecipitation assay buffer (50 mM Tris-HCl [pH 7.6], 150 mM NaCl, 1 mM EDTA, 1% Triton X-100, and 1 mM phenylmethylsulfonyl fluoride), and the protein lysates were resolved on 8% to 12% polyacrylamide gel. After electrophoresis, the proteins in the gel were transferred onto a polyvinylidene difluoride (PVDF) membrane (Bio-Rad) and were probed with specific antibodies. The rabbit polyclonal antibody that specifically recognizes GII-NTPase was generated in our laboratory using bacterially produced GII-NTPase(49–366) as an antigen. Antibodies to FLAG (A8592; Sigma), myc (sc-40 or sc-789; Santa Cruz), PARP (9532; Cell Signaling), cleaved caspase-3 (9664; Cell Signaling), caspase 3 (9662; Cell signaling), LC3B (NB100-2220; Novus Biologicals), GRP78 (sc-13968; Santa Cruz), and actin (sc-47778; Santa Cruz) were purchased commercially.

### Confocal immunofluorescence analysis.

A7 melanoma cells (1.5 × 10^5^) were seeded on coverslips in 6-well tissue culture plates and transiently transfected with the expression plasmids. After 24 h of transfection, cells were fixed with 4% paraformaldehyde in phosphate-buffered saline (PBS) at room temperature for 10 min and then washed with PBS three times for 5 min each. For mitochondrial staining, live cells were incubated with 250 nM MitoTracker (M7512; Thermo Fisher Scientific) at 37°C for 15 to 20 min prior to cell fixation. For immunofluorescence staining, the fixed cells were permeabilized with 0.1% Triton X-100 in PBS for 8 min at room temperature. Following a rinse with PBS, the cells were incubated with blocking solution (CAS-Block; Invitrogen) at room temperature for 30 min and then were treated with specific primary antibodies for 1 h. The primary antibodies against FLAG (A8592; Sigma), PDI (MA3-019; Thermo Fisher Scientific), GM130 (610822; BD Transduction Laboratories), TGN46 (NBP1-49643; Novus Biologicals), and myc (sc-40; Santa Cruz) were obtained commercially. After three further washing steps, appropriate secondary antibodies were added to treat cell samples, which included Alexa Fluor 488-conjugated goat-anti-mouse IgG (A-11001; Thermo Fisher Scientific) or goat-anti-rabbit IgG antibody (A-11008; Thermo Fisher Scientific), Alexa Fluor 633-conjugated goat-anti-mouse IgG antibody (A-21052; Thermo Fisher Scientific), or Alexa Fluor 594-conjugated goat-anti-mouse IgG antibody (A-11005; Thermo Fisher Scientific). Staining with 4′-6-diamidino-2-phenylindole (DAPI) was performed at room temperature for 15 min. Cells were mounted in CitiFluor (Agar Scientific) and were examined with a Leica confocal laser scanning system (TCS-SP5II). Image processing was carried out using LAS AF Lite software (Leica).

### Coimmunoprecipitation.

Coimmunoprecipitation analysis was performed as described previously (42). Briefly, 293T cells were cotransfected with expression vectors encoding myc-tagged NTPase and FLAG-tagged proteins as indicated in Fig. 6 to 8. After 24 h of transfection, the transfected cells were harvested and lysed in the immunoprecipitation assay buffer (50 mM Tris-HCl [pH 7.6], 150 mM NaCl, 1 mM EDTA, 1% Triton X-100, and 1 mM phenylmethylsulfonyl fluoride). Protein lysates were immunoprecipitated using anti-c-Myc agarose conjugate (A740; Sigma-Aldrich) or anti-FLAG magnetic beads (M8823; Sigma-Aldrich). After extensive washing with immunoprecipitation washing buffer (50 mM Tris-HCl [pH 7.6], 150 mM NaCl, 1 mM EDTA, 0.5% NP-40, and 10% glycerol), the immunoprecipitated proteins were analyzed by immunoblotting with specific antibodies.

### Accession number(s).

The GenBank accession numbers for the full-length viral genomes of the GII.4 and GI HuNV strains used in the study are MG049692 and MG049693, respectively.

## References

[1] Robilotti E, Deresinski S, Pinsky BA 2015 Norovirus. Clin Microbiol Rev 28:134–164. doi:10.1128/CMR.00075-14. 25567225 PMC4284304

[2] Rocha-Pereira J, Van Dycke J, Neyts J 2016 Norovirus genetic diversity and evolution: implications for antiviral therapy. Curr Opin Virol 20:92–98. doi:10.1016/j.coviro.2016.09.009. 27736665

[3] Kirby A, Iturriza-Gomara M 2012 Norovirus diagnostics: options, applications and interpretations. Expert Rev Anti Infect Ther 10:423–433. doi:10.1586/eri.12.21. 22512752

[4] Bok K, Abente EJ, Realpe-Quintero M, Mitra T, Sosnovtsev SV, Kapikian AZ, Green KY 2009 Evolutionary dynamics of GII.4 noroviruses over a 34-year period. J Virol 83:11890–11901. doi:10.1128/JVI.00864-09. 19759138 PMC2772697

[5] Boon D, Mahar JE, Abente EJ, Kirkwood CD, Purcell RH, Kapikian AZ, Green KY, Bok K 2011 Comparative evolution of GII.3 and GII.4 norovirus over a 31-year period. J Virol 85:8656–8666. doi:10.1128/JVI.00472-11. 21715504 PMC3165818

[6] Cannon JL, Lindesmith LC, Donaldson EF, Saxe L, Baric RS, Vinje J 2009 Herd immunity to GII.4 noroviruses is supported by outbreak patient sera. J Virol 83:5363–5374. doi:10.1128/JVI.02518-08. 19297483 PMC2681945

[7] Sarvestani ST, Cotton B, Fritzlar S, O'Donnell TB, Mackenzie JM 2016 Norovirus infection: replication, manipulation of host, and interaction with the host immune response. J Interferon Cytokine Res 36:215–225. doi:10.1089/jir.2015.0124. 27046239

[8] Jones MK, Watanabe M, Zhu S, Graves CL, Keyes LR, Grau KR, Gonzalez-Hernandez MB, Iovine NM, Wobus CE, Vinje J, Tibbetts SA, Wallet SM, Karst SM 2014 Enteric bacteria promote human and mouse norovirus infection of B cells. Science 346:755–759. doi:10.1126/science.1257147. 25378626 PMC4401463

[9] Ettayebi K, Crawford SE, Murakami K, Broughman JR, Karandikar U, Tenge VR, Neill FH, Blutt SE, Zeng XL, Qu L, Kou B, Opekun AR, Burrin D, Graham DY, Ramani S, Atmar RL, Estes MK 2016 Replication of human noroviruses in stem cell-derived human enteroids. Science 353:1387–1393. doi:10.1126/science.aaf5211. 27562956 PMC5305121

[10] Katayama K, Murakami K, Sharp TM, Guix S, Oka T, Takai-Todaka R, Nakanishi A, Crawford SE, Atmar RL, Estes MK 2014 Plasmid-based human norovirus reverse genetics system produces reporter-tagged progeny virus containing infectious genomic RNA. Proc Natl Acad Sci U S A 111:E4043–E4052. doi:10.1073/pnas.1415096111. 25192933 PMC4183323

[11] Asanaka M, Atmar RL, Ruvolo V, Crawford SE, Neill FH, Estes MK 2005 Replication and packaging of Norwalk virus RNA in cultured mammalian cells. Proc Natl Acad Sci U S A 102:10327–10332. doi:10.1073/pnas.0408529102. 16002473 PMC1177355

[12] Donaldson EF, Lindesmith LC, Lobue AD, Baric RS 2008 Norovirus pathogenesis: mechanisms of persistence and immune evasion in human populations. Immunol Rev 225:190–211. doi:10.1111/j.1600-065X.2008.00680.x. 18837783

[13] Wobus CE, Karst SM, Thackray LB, Chang KO, Sosnovtsev SV, Belliot G, Krug A, Mackenzie JM, Green KY, Virgin HW 2004 Replication of norovirus in cell culture reveals a tropism for dendritic cells and macrophages. PLoS Biol 2:e432. doi:10.1371/journal.pbio.0020432. 15562321 PMC532393

[14] Thorne LG, Goodfellow IG 2014 Norovirus gene expression and replication. J Gen Virol 95:278–291. doi:10.1099/vir.0.059634-0. 24243731

[15] Ettayebi K, Hardy ME 2003 Norwalk virus nonstructural protein p48 forms a complex with the SNARE regulator VAP-A and prevents cell surface expression of vesicular stomatitis virus G protein. J Virol 77:11790–11797. doi:10.1128/JVI.77.21.11790-11797.2003. 14557663 PMC229264

[16] Fernandez-Vega V, Sosnovtsev SV, Belliot G, King AD, Mitra T, Gorbalenya A, Green KY 2004 Norwalk virus N-terminal nonstructural protein is associated with disassembly of the Golgi complex in transfected cells. J Virol 78:4827–4837. doi:10.1128/JVI.78.9.4827-4837.2004. 15078964 PMC387691

[17] Sharp TM, Crawford SE, Ajami NJ, Neill FH, Atmar RL, Katayama K, Utama B, Estes MK 2012 Secretory pathway antagonism by calicivirus homologues of Norwalk virus nonstructural protein p22 is restricted to noroviruses. Virol J 9:181. doi:10.1186/1743-422X-9-181. 22943503 PMC3493335

[18] Sharp TM, Guix S, Katayama K, Crawford SE, Estes MK 2010 Inhibition of cellular protein secretion by Norwalk virus nonstructural protein p22 requires a mimic of an endoplasmic reticulum export signal. PLoS One 5:e13130. doi:10.1371/journal.pone.0013130. 20976190 PMC2956632

[19] Pfister T, Wimmer E 2001 Polypeptide p41 of a Norwalk-like virus is a nucleic acid-independent nucleoside triphosphatase. J Virol 75:1611–1619. doi:10.1128/JVI.75.4.1611-1619.2001. 11160659 PMC114070

[20] Cotton BT, Hyde JL, Sarvestani ST, Sosnovtsev SV, Green KY, White PA, Mackenzie JM 2017 The norovirus NS3 protein is a dynamic lipid- and microtubule-associated protein involved in viral RNA replication. J Virol 91:e02138-16. doi:10.1128/JVI.02138-16. 27881660 PMC5244318

[21] Hyde JL, Mackenzie JM 2010 Subcellular localization of the MNV-1 ORF1 proteins and their potential roles in the formation of the MNV-1 replication complex. Virology 406:138–148. doi:10.1016/j.virol.2010.06.047. 20674956

[22] Chang KO, Sosnovtsev SV, Belliot G, King AD, Green KY 2006 Stable expression of a Norwalk virus RNA replicon in a human hepatoma cell line. Virology 353:463–473. doi:10.1016/j.virol.2006.06.006. 16843517

[23] Guix S, Asanaka M, Katayama K, Crawford SE, Neill FH, Atmar RL, Estes MK 2007 Norwalk virus RNA is infectious in mammalian cells. J Virol 81:12238–12248. doi:10.1128/JVI.01489-07. 17855551 PMC2168986

[24] Hyde JL, Sosnovtsev SV, Green KY, Wobus C, Virgin HW, Mackenzie JM 2009 Mouse norovirus replication is associated with virus-induced vesicle clusters originating from membranes derived from the secretory pathway. J Virol 83:9709–9719. doi:10.1128/JVI.00600-09. 19587041 PMC2748037

[25] Hyde JL, Gillespie LK, Mackenzie JM 2012 Mouse norovirus 1 utilizes the cytoskeleton network to establish localization of the replication complex proximal to the microtubule organizing center. J Virol 86:4110–4122. doi:10.1128/JVI.05784-11. 22301146 PMC3318650

[26] Yalamanchili P, Datta U, Dasgupta A 1997 Inhibition of host cell transcription by poliovirus: cleavage of transcription factor CREB by poliovirus-encoded protease 3Cpro. J Virol 71:1220–1226. 8995645 10.1128/jvi.71.2.1220-1226.1997 PMC191176

[27] Weidman MK, Yalamanchili P, Ng B, Tsai W, Dasgupta A 2001 Poliovirus 3C protease-mediated degradation of transcriptional activator p53 requires a cellular activity. Virology 291:260–271. doi:10.1006/viro.2001.1215. 11878895

[28] Weng KF, Li ML, Hung CT, Shih SR 2009 Enterovirus 71 3C protease cleaves a novel target CstF-64 and inhibits cellular polyadenylation. PLoS Pathog 5:e1000593. doi:10.1371/journal.ppat.1000593. 19779565 PMC2742901

[29] Liu YC, Kuo RL, Lin JY, Huang PN, Huang Y, Liu H, Arnold JJ, Chen SJ, Wang RY, Cameron CE, Shih SR 2014 Cytoplasmic viral RNA-dependent RNA polymerase disrupts the intracellular splicing machinery by entering the nucleus and interfering with Prp8. PLoS Pathog 10:e1004199. doi:10.1371/journal.ppat.1004199. 24968230 PMC4072778

[30] Mitra K, Wunder C, Roysam B, Lin G, Lippincott-Schwartz J 2009 A hyperfused mitochondrial state achieved at G1-S regulates cyclin E buildup and entry into S phase. Proc Natl Acad Sci U S A 106:11960–11965. doi:10.1073/pnas.0904875106. 19617534 PMC2710990

[31] Peng JY, Lin CC, Chen YJ, Kao LS, Liu YC, Chou CC, Huang YH, Chang FR, Wu YC, Tsai YS, Hsu CN 2011 Automatic morphological subtyping reveals new roles of caspases in mitochondrial dynamics. PLoS Comput Biol 7:e1002212. doi:10.1371/journal.pcbi.1002212. 21998575 PMC3188504

[32] Wai T, Langer T 2016 Mitochondrial dynamics and metabolic regulation. Trends Endocrinol Metab 27:105–117. doi:10.1016/j.tem.2015.12.001. 26754340

[33] Finn RD, Attwood TK, Babbitt PC, Bateman A, Bork P, Bridge AJ, Chang HY, Dosztanyi Z, El-Gebali S, Fraser M, Gough J, Haft D, Holliday GL, Huang H, Huang X, Letunic I, Lopez R, Lu S, Marchler-Bauer A, Mi H, Mistry J, Natale DA, Necci M, Nuka G, Orengo CA, Park Y, Pesseat S, Piovesan D, Potter SC, Rawlings ND, Redaschi N, Richardson L, Rivoire C, Sangrador-Vegas A, Sigrist C, Sillitoe I, Smithers B, Squizzato S, Sutton G, Thanki N, Thomas PD, Tosatto SC, Wu CH, Xenarios I, Yeh LS, Young SY, Mitchell AL 2017 InterPro in 2017-beyond protein family and domain annotations. Nucleic Acids Res 45:D190–D199. doi:10.1093/nar/gkw1107. 27899635 PMC5210578

[34] Gao Q, Goodman JM 2015 The lipid droplet–a well-connected organelle. Front Cell Dev Biol 3:49. doi:10.3389/fcell.2015.00049. 26322308 PMC4533013

[35] Zhang S, Wang Y, Cui L, Deng Y, Xu S, Yu J, Cichello S, Serrero G, Ying Y, Liu P 2016 Morphologically and functionally distinct lipid droplet subpopulations. Sci Rep 6:29539. doi:10.1038/srep29539. 27386790 PMC4937419

[36] Bok K, Prikhodko VG, Green KY, Sosnovtsev SV 2009 Apoptosis in murine norovirus-infected RAW264.7 cells is associated with downregulation of survivin. J Virol 83:3647–3656. doi:10.1128/JVI.02028-08. 19211757 PMC2663291

[37] Herod MR, Salim O, Skilton RJ, Prince CA, Ward VK, Lambden PR, Clarke IN 2014 Expression of the murine norovirus (MNV) ORF1 polyprotein is sufficient to induce apoptosis in a virus-free cell model. PLoS One 9:e90679. doi:10.1371/journal.pone.0090679. 24599381 PMC3944349

[38] Graham FL, Smiley J, Russell WC, Nairn R 1977 Characteristics of a human cell line transformed by DNA from human adenovirus type 5. J Gen Virol 36:59–74. 886304 10.1099/0022-1317-36-1-59

[39] Chiu YF, Sugden B, Chang PJ, Chen LW, Lin YJ, Lan YC, Lai CH, Liou JY, Liu ST, Hung CH 2012 Characterization and intracellular trafficking of Epstein-Barr virus BBLF1, a protein involved in virion maturation. J Virol 86:9647–9655. doi:10.1128/JVI.01126-12. 22740416 PMC3446546

[40] Bryksin AV, Matsumura I 2010 Overlap extension PCR cloning: a simple and reliable way to create recombinant plasmids. Biotechniques 48:463–465. doi:10.2144/000113418. 20569222 PMC3121328

[41] Chang PJ, Chen LW, Chen LY, Hung CH, Shih YJ, Wang SS 12 7 2017 Effects of the NEDD8-activating enzyme inhibitor MLN4924 on lytic reactivation of Kaposi's sarcoma-associated herpesvirus. J Virol doi:10.1128/JVI.00505-17. PMC5599746 28701396

[42] Chang TH, Wang SS, Chen LW, Shih YJ, Chang LK, Liu ST, Chang PJ 2016 Regulation of the abundance of Kaposi's sarcoma-associated herpesvirus ORF50 protein by oncoprotein MDM2. PLoS Pathog 12:e1005918. doi:10.1371/journal.ppat.1005918. 27698494 PMC5047794

